# Association between the newly proposed dietary index for gut microbiota and thyroid function: NHANES 2007–2012

**DOI:** 10.3389/fnut.2025.1602787

**Published:** 2025-06-26

**Authors:** Ke Peng, Hanjie Guo, Zhiqiang Zhang, Weidong Xiao

**Affiliations:** Department of General Surgery, Xinqiao Hospital, Army Medical University, Chongqing, China

**Keywords:** dietary index for gut microbiota, thyroid function, gut microbiota, National Health and Nutrition Examination Survey, cross-sectional study

## Abstract

**Aims:**

Diet-gut-thyroid axis has attracted increasing interest. The dietary index for gut microbiota (DI-GM) is a recently introduced measure of diet quality that represents gut microbiota diversity. However, its relationship with thyroid function has not been investigated. This study aimed to examine the unexplored relationship between DI-GM and thyroid function.

**Methods:**

This cross-sectional study analyzed data from 6,126 participants aged ≥20 years in the National Health and Nutrition Examination Survey (NHANES). Linear regression models, smoothed curve fitting, and subgroup analyses were used to examine the relationship between DI-GM and thyroid function.

**Results:**

After controlling for all covariates, higher DI-GM scores were significantly associated with lower levels of free triiodothyronine (FT3) (*β* = −0.043, 95% CI = −0.077, −0.010, *P* for trend = 0.010), free thyroxine (FT4) (*β* = −0.011, 95% CI = −0.021, −0.002, *P* for trend = 0.027), and total thyroxine (TT4) (*β* = −0.127, 95% CI = −0.237, −0.017, *P* for trend = 0.024). Smooth curve fitting analysis confirmed a linear relationship between DI-GM and FT3, FT4, and TT4 levels. Furthermore, subgroup analyses indicated that age may influence the negative correlation between DI-GM and FT4 levels, with participants aged < 40 years exhibiting a more pronounced decrease in FT4 levels (*β* = −0.090, 95% CI: −0.140, −0.041). Smoking status may modify the relationship between DI-GM and thyroid hormone levels, showing negative correlations for FT3 levels only in never smokers (*β* = −0.073, 95% CI = −0.114, −0.032) and for TT4 levels only in former smokers (β = −0.316, 95% CI = −0.511, −0.122). Notably, thyroglobulin antibodies levels exhibited opposing directional effects between never smokers (negative) and former/current smokers (positive).

**Conclusion:**

Higher DI-GM scores were negatively correlated with lower FT3, FT4, and TT4 levels, with age and smoking status serving as key factors influencing this association.

## 1 Introduction

The thyroid gland, the body’s largest endocrine gland, is responsible for the synthesis and secretion of thyroid hormones, thyroxine (T4) and triiodothyronine (T3). Thyroid hormones play a crucial role in brain development, metabolic, mood regulation and other physiological functions (1). Studies indicated that abnormal thyroid hormone levels, both deficiency and excess, can lead to adverse health effects. Thyroid hormones deficiency increased the risk of atherosclerosis, ischemic heart disease (2, 3), and non-alcoholic fatty liver disease (4). Whereas thyroid hormones excess givers rise to atrial fibrillation, heart failure (2, 3) and osteoporosis (5). In addition to non-modifiable factors such as gender, age and genetics, dietary is a important modifiable risk factor that can influence thyroid hormone levels. Recent studies have highlighted that a multitude of dietary factors from dietary protein to macro and micronutrients intakes are critical for thyroid gland function, in metabolism and in synthesis of thyroid hormones (6–10). It is therefore essential develop a detailed understanding relationship between diet and thyroid hormones.

The gut microbiome consists of trillions of microorganisms that have an impact on human health by extracting nutrients from food and producing metabolites that can influence human metabolic processes. Increasing evidence (7, 11) has indicated that dietary is closely associated with thyroid function through the the gut–thyroid axis. An imbalance in gut microbiota can impair the intestinal immune system, enhance intestinal permeability, and facilitate bacterial translocation, resulting in systemic and local tissue inflammation that can ultimately affect thyroid hormone levels (12). Gut microbiota can metabolize primary bile acids into secondary bile acids in the small intestine, thereby increasing the activity of deiodinase enzyme, promoting the conversion of T4 to T3 and inhibiting the secretion of pituitary thyroid-stimulating hormone (TSH) (13). Furthermore, the gut microbiota plays a key role in regulating the availability of essential micronutrients for thyroid function, such as iodine, selenium, zinc, and iron. Lipopolysaccharides (LPS) and short-chain fatty acids (SCFAs) produced by the gut microbiota can modulate iodine uptake by affecting the expression and activity of sodium-iodide symporter (14). Moreover, SCFAs can lower intestinal pH and enhance the bioavailability of colonic iron (14, 15). Gut microbiota produce and secrete siderophores with high affinity for Fe(III), such as enterobactin, to mediate iron uptake (15). Some Lactic Acid Bacteria are able to fix inorganic selenite into seleno amino acids (16). The transformation from inorganic to organic states increases the bioavailability of selenium (17).

Despite diet can affect gut microbiota composition (18), there is a lack of a comprehensive measure of diet or a dietary index that can quantify individuals’ diets in terms of attaining a healthy gut microbiota. To fill the gap in this field, Kase et al. (19) identified 14 dietary components associated with gut microbiota, through a comprehensive literature review on the relationship between diet and gut microbiota in adults. Among these, foods such as chickpeas, soybeans, whole grains, fiber, cranberries, fermented dairy products, avocados, broccoli, coffee, and green tea were found to have beneficial effects on gut microbiota. In contrast, red meat, processed meat, refined grains, and high-fat diets negatively impact gut microbial health. The researchers subsequently developed and evaluated a novel dietary index for gut microbiota (DI-GM) by using the dietary data of the National Health and Nutrition Examination Survey (NHANES) of the United States (19). Unlike existing dietary assessment tools, the DI-GM provides a more comprehensive assessment of the relationship between diet and gut microbiota. DI-GM reflects changes in gut microbiota diversity, levels of SCFA production, and changes in certain specific bacterial phyla (19). As promising standardized tool for evaluating balanced diets that support gut microbiota, DI-GM has been extensively studied in various diseases, such as diabetes (20), non-alcoholic fatty liver disease (21), metabolic syndrome (22) and sarcopenia (23). However, the relationship between DI-GM and thyroid function remains poorly explored.

This study involved a cross-sectional analysis of the associations between DI-GM and thyroid function using a large sample of individuals aged 20 years or older from the 2007–2012 NHANES data. After a comprehensive and rigorous analysis, we aim to reveal the potential of DI-GM as a dietary quality indicator in predicting thyroid function, and provide new theoretical insights for future dietary intervention strategies.

## 2 Materials and methods

### 2.1 Study design and population

This study used data from the NHANES, which covers three cycles from 2007 to 2012. NHANES is a nationwide continuous cross-sectional biennial survey to assess the health and nutritional status of adults and children in the United States. The survey protocols were approved by the Institutional Review Board of the National Center for Health Statistics at the Centers for Disease Control and Prevention. All participants provided informed written consent before participating in the survey. All data and materials are publicly available for researchers at https://www.cdc.gov/nchs/nhanes/.

**Figure 1:**
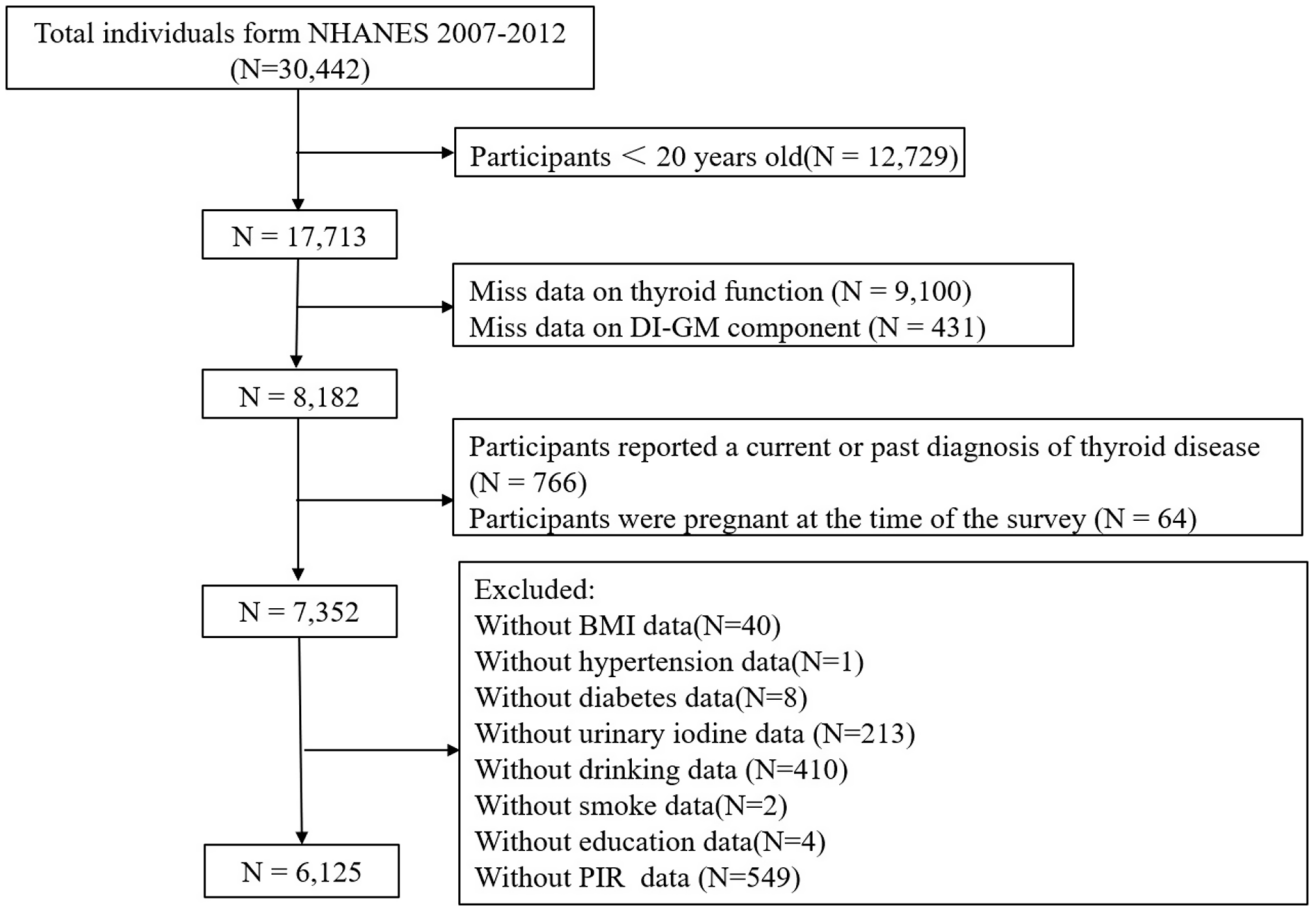
Flow chart of the current study.

This study involved a total of 30,442 participants from 2007 to 2012. The inclusion criteria were as follows: (1) participants aged 20 years or older; (2) available DI-GM data; (3) complete data on thyroid function parameters. The exclusion criteria were as follows: (1) participants reported a current or past diagnosis of thyroid disease; (2) pregnant women; (3) missing or incomplete covariate data, such as body mass index (BMI), hypertension, diabetes, urinary iodine, alcohol consumption, smoking, education level, and poverty income ratio (PIR). Ultimately 6,126 participants were included in the final analysis, as shown in Figure 1.

### 2.2 Assessment of thyroid function

This study evaluated thyroid function parameters, including total and free thyroxine (TT4 and FT4), total and free triiodothyronine (TT3 and FT3), thyroglobulin (Tg), thyroglobulin antibodies (TgAb), thyroid peroxidase antibodies (TPOAb), and TSH. Detailed procedures for serum specimen collection and processing are outlined in the NHANES Laboratory/Medical Technologists Procedures Manual (LPM), accessible at: https://wwwn.cdc.gov/Nchs/Data/Nhanes/Public/2007/DataFiles/THYROD_E.htm).

### 2.3 Assessment of the dietary index for gut microbiota

Kase et al. (19) conducted a review of 106 articles and identified 14 dietary components that contribute to the DI-GM score. Beneficial components included fermented dairy, chickpeas, soybean, whole grains, fiber, cranberries, avocados, broccoli, coffee, and green tea, while unfavorable components comprised red meat, processed meat, refined grains, and a high-fat diet (≥40% of total energy from fat). For beneficial foods, participants consuming amounts above the sex-specific median were assigned a score of 1, whereas those with intake below the median were assigned a score of 0. Conversely, for unfavorable foods, participants consuming amounts above the sex-specific median receive a score of 0, whereas those below the median receive a score of 1. The total DI-GM score, calculated by summing the individual component scores, ranges from 0 to 13 and is categorized into four groups: 0–3, 4, 5, and ≥6. More detailed information about the composition and calculation of DI-GM can be found in Supplementary Table 1.

### 2.4 Covariates

To account for potential confounders, numerous covariates were incorporated into the analysis. These covariates included age, sex (male, female), ethnicity (Mexican American, non-Hispanic White, non-Hispanic Black, other Hispanic, and other races), educational level (below high school, high school or equivalent, college graduate or higher), PIR, smoking status, alcohol consumption, urine iodine concentration, BMI, hypertension, and diabetes. These covariates were selected based on their established association with thyroid function and dietary patterns (24, 25).

PIR, calculated as the ratio of family income to the poverty threshold set by the US Census Bureau, was classified into three categories: ≤1.3, 1.3–3.5, and >3.5, representing different levels of socioeconomic status among participants (26). According to the NHANES guidelines, smoking status was classified as “Never,” “Now,” and “Former.” Individuals who had smoked fewer than 100 cigarettes in their lifetime were categorized as “Never” smokers, whereas those who had smoked more than 100 cigarettes but were not currently smoking were classified as “Former” smokers (26). Similarly, alcohol consumption was categorized as never, former, or current use. Current alcohol users were further classified into three groups: (1) heavy drinkers (≥ 3 drinks per day or binge drinking [≥ 4 drinks per occasion] on ≥ 5 days per month for females, ≥ 4 drinks per day or binge drinking [≥ 5 drinks per occasion] on ≥ 5 days per month for males; (2) moderate drinkers (≥ 2 drinks per day for females, ≥ 3 drinks per day for males, or binge drinking on ≥ 2 days per month); and (3) mild drinkers, who consumed alcohol but did not meet the criteria for heavy or moderate drinking (27). BMI was calculated by dividing body weight (kg) by the square of height (m). Hypertension was identified based on a self-reported diagnosis, use of antihypertensive medication, or measured SBP of ≥ 140 mm Hg or DBP of ≥ 90 mm Hg. Diabetes mellitus was classified based on a self-reported diagnosis by a physician or health professional or the use of antidiabetic medications.

### 2.5 Statistical analysis

In this study, categorical variables were presented as frequencies (N) and percentages (%), whereas continuous variables were reported as mean ± SD or medians (Q1, Q3). Categorical variables were analyzed using the Chi-square test to compare differences between groups. For continuous variables, one-way ANOVA was applied to those with normal distribution, whereas the Kruskal–Wallis H test was used for skewed distributions.

DI-GM was examined as a continuous and categorical variable. To mitigate the impact of non-normal distribution, a logarithmic transformation was applied to DI-GM before analysis. A multivariate linear regression analysis was performed to assess the relationship between DI-GM and thyroid function indicators. Three models were used: Model 1 was adjusted for age, sex, and ethnicity; Model 2 included additional adjustments for education level, PIR, smoking status, alcohol consumption, and BMI; and Model 3 accounted for all covariates. Trend analyses (*P* for trend) were conducted by treating the DI-GM grouping variable as a continuous variable and reapplying the corresponding regression models. In addition, spline smoothing using a generalized additive model (GAM) was performed to visually illustrate the correlation between DI-GM and thyroid function indicators. Additionally, a threshold analysis was conducted to explore whether there was a significant inflection point using piecewise linear regression models. Finally, subgroup analyses were conducted according to age, sex, BMI, and smoking status.

The data analysis was conducted using R software (The R foundation; version 4.2.0) and EmopwerStats (www.empowerstats.net, X&Y solutions, Inc., Boston, Massachusetts).

## 3 Results

### 3.1 Baseline characteristics of participants

This study included 6,125 participants. Participants with higher DI-GM scores exhibited a higher mean age, a greater proportion of females and non-Hispanic White people, higher education levels, increased PIR levels, lower BMI, and a lower prevalence of smoking and alcohol consumption than participants with lower DI-GM scores (0–3). Furthermore, they exhibited lower levels of FT3, TT3, and TT4. Table 1 presents the baseline characteristics of the participants.

**Table 1:** Participant baseline characteristics.

Variable	Total (*N* = 6,125)	DI-GM				*P* Value
		0–3 (*N* = 1,233)	4 (*N* = 1,308)	5 (*N* = 1,425)	≥6 (*N* = 2,159)	
Age	48.0 (34.0–63.0)	46.0 (32.0–61.0)	47.0 (33.0–62.0)	47.0 (33.0–63.0)	51.0 (36.5–64.0)	<0.001
Age						<0.001
20–39	2,151 (35.12%)	494 (40.06%)	483 (36.93%)	515 (36.14%)	659 (30.52%)	
40–59	2023 (33.03%)	404 (32.77%)	423 (32.34%)	461 (32.35%)	735 (34.04%)	
≥60	1951 (31.85%)	335 (27.17%)	402 (30.73%)	449 (31.51%)	765 (35.43%)	
Gender						<0.001
Male	3,316 (54.14%)	718 (58.23%)	744 (56.88%)	748 (52.49%)	1,106 (51.23%)	
Female	2,809 (45.86%)	515 (41.77%)	564 (43.12%)	677 (47.51%)	1,053 (48.77%)	
Race						<0.001
Mexican American	969 (15.82%)	202 (16.38%)	247 (18.88%)	247 (17.33%)	273 (12.64%)	
Non-Hispanic White	2,873 (46.91%)	519 (42.09%)	554 (42.35%)	631 (44.28%)	1,169 (54.15%)	
Non-Hispanic Black	1,265 (20.65%)	328 (26.60%)	280 (21.41%)	301 (21.12%)	356 (16.49%)	
Other Hispanic	639 (10.43%)	127 (10.30%)	151 (11.54%)	158 (11.09%)	203 (9.40%)	
Other race	379 (6.19%)	57 (4.62%)	76 (5.81%)	88 (6.18%)	158 (7.32%)	
PIR						<0.001
≤1.3	1915 (31.27%)	469 (38.04%)	455 (34.79%)	477 (33.47%)	514 (23.81%)	
1.3–3.5	2,313 (37.76%)	463 (37.55%)	514 (39.30%)	531 (37.26%)	805 (37.29%)	
>3.5	1897 (30.97%)	301 (24.41%)	339 (25.92%)	417 (29.26%)	840 (38.91%)	
Education						<0.001
Under high school	1705 (27.84%)	423 (34.31%)	432 (33.03%)	412 (28.91%)	438 (20.29%)	
High school or equivalent	1,423 (23.23%)	327 (26.52%)	314 (24.01%)	349 (24.49%)	433 (20.06%)	
College graduate or above	2,997 (48.93%)	483 (39.17%)	562 (42.97%)	664 (46.60%)	1,288 (59.66%)	
TSH	1.55 (1.05–2.29)	1.51 (1.02–2.24)	1.50 (1.03–2.24)	1.57 (1.08–2.27)	1.60 (1.08–2.35)	0.150
FT3	3.19 ± 0.50	3.25 ± 0.82	3.20 ± 0.39	3.19 ± 0.38	3.14 ± 0.37	<0.001
FT4	0.79 ± 0.14	0.80 ± 0.17	0.80 ± 0.14	0.79 ± 0.13	0.79 ± 0.13	0.053
TT3	113.80 ± 22.83	114.18 ± 21.84	114.32 ± 23.38	115.10 ± 24.22	112.41 ± 22.04	0.004
TT4	7.86 ± 1.58	7.95 ± 1.58	7.90 ± 1.68	7.85 ± 1.48	7.79 ± 1.57	0.032
TgAb	0.60 (0.60–0.60)	0.60 (0.60–0.60)	0.60 (0.60–0.60)	0.60 (0.60–0.60)	0.60 (0.60–0.60)	0.778
TPOAb	0.60 (0.30–1.40)	0.60 (0.30–1.30)	0.60 (0.30–1.40)	0.60 (0.30–1.20)	0.60 (0.30–1.50)	0.743
Tg	10.28 (5.98–17.66)	10.56 (6.32–18.53)	10.07 (5.91–17.46)	10.86 (6.12–17.82)	9.86 (5.75–17.05)	0.438
Urine iodine	146.90 (83.80–249.70)	148.60 (83.70–255.80)	150.80 (89.68–251.98)	149.80 (85.60–255.40)	142.80 (80.65–239.90)	0.506
BMI	27.90 (24.32–32.24)	28.03 (24.61–32.71)	27.98 (24.06–32.30)	28.17 (24.56–32.59)	27.60 (24.20–31.70)	0.005
Smoke						<0.001
Never	3,186 (52.02%)	637 (51.66%)	644 (49.24%)	752 (52.77%)	1,153 (53.40%)	
Former	1,574 (25.70%)	276 (22.38%)	339 (25.92%)	356 (24.98%)	603 (27.93%)	
Now	1,365 (22.29%)	320 (25.95%)	325 (24.85%)	317 (22.25%)	403 (18.67%)	
Alcohol user						<0.001
Never	788 (12.87%)	151 (12.25%)	185 (14.14%)	191 (13.40%)	261 (12.09%)	
Former	1,154 (18.84%)	253 (20.52%)	238 (18.20%)	276 (19.37%)	387 (17.92%)	
Mild	1922 (31.38%)	327 (26.52%)	368 (28.13%)	431 (30.25%)	796 (36.87%)	
Moderate	929 (15.17%)	178 (14.44%)	191 (14.60%)	218 (15.30%)	342 (15.84%)	
Heavy	1,332 (21.75%)	324 (26.28%)	326 (24.92%)	309 (21.68%)	373 (17.28%)	
Hypertension						0.671
No	3,622 (59.13%)	712 (57.75%)	771 (58.94%)	855 (60.00%)	1,284 (59.47%)	
Yes	2,503 (40.87%)	521 (42.25%)	537 (41.06%)	570 (40.00%)	875 (40.53%)	
Diabetes						0.140
No	5,372 (87.71%)	1,058 (85.81%)	1,154 (88.23%)	1,251 (87.79%)	1909 (88.42%)	
Yes	753 (12.29%)	175 (14.19%)	154 (11.77%)	174 (12.21%)	250 (11.58%)	

### 3.2 Association between DI-GM and serum thyroid function indicators

Multivariate linear regression analysis (Table 2) revealed a significant negative association between the DI-GM score and FT3 (Model 1: *β* = −0.056, 95% CI = −0.085, −0.026; Model 2: β = −0.038, 95% CI = −0.068, −0.008; and Model 3: β = −0.039, 95% CI = −0.069, −0.008), FT4 (Model 1: β = −0.010, 95% CI = −0.019, −0.001; Model 2: β = −0.011, 95% CI = −0.019, −0.002; and Model 3: β = −0.010, 95% CI = −0.019, −0.001), and TT4 (Model 1: β = −0.163, 95% CI = −0.261, −0.065; Model 2: β = −0.130, 95% CI = −0.229, −0.032; and Model 3: *β* = −0.123, 95% CI = −0.222, −0.025). While statistically significant, the observed *β* coefficients suggest a modest change in thyroid hormone levels per unit increase in DI-GM. Specifically, there was 0.039 pg/mL, 0.01 ng/dL and 0.222 μg/dL reduction in FT3, FT4 and TT4 levels per each unit increase in DI-GM. However, no statistically significant associations were observed for TSH, TT3, TPOAb, TgAb, and Tg.

**Table 2:** Association between the newly proposed dietary index for gut microbiota and thyroid function.

Variable	Model 1 *β* (95%CI)	Model 2 *β* (95%CI)	Model 3 *β* (95%CI)
TSH (mIU/L)			
DI-GM	−0.030 (−0.156, 0.097)	−0.029 (−0.157, 0.100)	−0.028 (−0.157, 0.101)
DI-GM group			
0–3	Ref.	Ref.	Ref.
4	−0.039 (−0.195, 0.118)	−0.030 (−0.187, 0.126)	−0.029 (−0.186, 0.128)
5	−0.028 (−0.181, 0.126)	−0.026 (−0.180, 0.128)	−0.025 (−0.178, 0.129)
≥6	0.031(−0.111, 0.173)	0.038 (−0.106, 0.182)	0.039 (−0.105, 0.183)
*P* for trend	0.708	0.298	0.640
Beneficial to gut microbiota	−0.011 (−0.110, 0.089)	−0.013 (−0.115, 0.089)	−0.015 (−0.117, 0.087)
Unfavorable to gut microbiota	−0.042 (−0.163, 0.078)	−0.030 (−0.150, 0.090)	−0.028 (−0.149, 0.092)
FT3 (pg/ml)			
DI-GM	−0.056 (−0.085, −0.026)	−0.038 (−0.068, −0.008)	−0.039 (−0.069, −0.008)
DI-GM group			
0–3	Ref.	Ref.	Ref.
4	−0.041 (−0.078, −0.004)	−0.036 (−0.072, 0.0008)	−0.037 (−0.074, −0.0006)
5	−0.045 (−0.081, −0.009)	−0.039 (−0.075, −0.003)	−0.041 (−0.076, −0.005)
≥6	−0.061 (−0.094, −0.028)	−0.042 (−0.076, −0.009)	−0.043 (−0.077, −0.010)
*P* for trend	<0.001	0.030	0.010
Beneficial to gut microbiota	−0.037 (−0.061, −0.013)	−0.022 (−0.047, 0.002)	−0.020 (−0.045, 0.004)
Unfavorable to gut microbiota	−0.038 (−0.066, −0.010)	−0.035 (−0.063, −0.007)	−0.037 (−0.065, −0.009)
FT4 (ng/dL)			
DI-GM	−0.010 (−0.019, −0.001)	−0.011 (−0.019, −0.002)	−0.010 (−0.019, −0.001)
DI-GM group			
0–3	Ref.	Ref.	Ref.
4	−0.00004 (−0.011, 0.011)	−0.0003 (−0.011,0.011)	0.0002 (−0.011, 0.011)
5	−0.011 (−0.022, −0.0006)	−0.012 (−0.022, −0.001)	−0.011 (−0.022, −0.0003)
≥6	−0.011 (−0.021, −0.002)	−0.012 (−0.022, −0.002)	−0.011 (−0.021, −0.002)
*P* for trend	0.026	0.042	0.027
Beneficial to gut microbiota	−0.004 (−0.011, 0.003)	−0.005 (−0.012, 0.002)	−0.005 (−0.012, 0.002)
Unfavorable to gut microbiota	0.006 (−0.002, 0.014)	0.005 (−0.004, 0.013)	0.005 (−0.003, 0.013)
TT3 (ng/dL)			
DI-GM	−0.052 (−1.435, 1.332)	1.020 (−0.373, 2.413)	0.930 (−0.461, 2.321)
DI-GM group			
0–3	Ref.	Ref.	Ref.
4	0.360 (−1.348, 2.068)	0.604 (−1.089, 2.297)	0.501 (−1.187, 2.190)
5	1.385 (−0.290, 3.060)	1.699 (0.037, 3.361)	1.582 (−0.076, 3.239)
≥6	−0.273 (−1.823,1.277)	0.777 (−0.781, 2.334)	0.672 (−0.882, 2.226)
*P* for trend	0.800	0.212	0.363
Beneficial to gut microbiota	−0.564 (−1.653, 0.524)	0.302 (−0.801, 1.406)	0.404 (−0.697, 1.505)
Unfavorable to gut microbiota	−0.224 (−1.523, 1.076)	−0.121 (−1.410, 1.169)	−0.235 (−1.522, 1.052)
TT4 (μg/dL)			
DI-GM	−0.163 (−0.261, −0.065)	−0.130 (−0.229, −0.032)	−0.123 (−0.222, −0.025)
DI-GM group			
0–3	Ref.	Ref.	Ref.
4	−0.071 (−0.192,0.05)	−0.062 (−0.182, 0.058)	−0.056 (−0.176, 0.064)
5	−0.133 (−0.251, −0.014)	−0.131 (−0.249, −0.013)	−0.123 (−0.241, −0.005)
≥6	−0.164 (−0.274, −0.054)	−0.135 (−0.245, −0.024)	−0.127 (−0.237, −0.017)
*P* for trend	0.004	0.013	0.024
Beneficial to gut microbiota	−0.058 (−0.134, 0.019)	−0.038 (−0.116, 0.039)	−0.042 (−0.120, 0.036)
Unfavorable to gut microbiota	0.022 (−0.070, 0.115)	0.028 (−0.064, 0.119)	0.035 (−0.057, 0.126)
TPOAb (IU/mL)			
DI-GM	0.154 (−5.174, 5.482)	−0.510 (−5.932, 4.911)	−0.667 (−6.092, 4.759)
DI-GM group			
0–3	Ref.	Ref.	Ref.
4	−0.289 (−6.866, 6.288)	−0.373 (−6.957, 6.212)	−0.286 (−6.871, 6.299)
5	1.499 (−4.952, 7.950)	1.136 (−5.327, 7.599)	1.078 (−5.386, 7.543)
≥6	1.200 (−4.770, 7.169)	0.554 (−5.503, 6.612)	0.463 (−5.597, 6.523)
*P* for trend	0.697	0.726	0.881
Beneficial to gut microbiota	0.429 (−3.705, 4.563)	−0.345 (−4.580, 3.890)	−0.605 (−4.844, 3.635)
Unfavorable to gut microbiota	−0.447 (−5.478, 4.583)	−0.031 (−5.076, 5.014)	0.156 (−4.892, 5.204)
TgAb (IU/mL)			
DI-GM	−0.563 (−5.125, 3.999)	−0.945 (−5.592, 3.702)	−1.036 (−5.688, 3.616)
DI-GM group			
0–3	Ref.	Ref.	Ref.
4	−1.555 (−7.186, 4.077)	−1.605 (−7.248, 4.039)	−1.673 (−7.319, 3.972)
5	0.308 (−5.215, 5.831)	0.139 (−5.400, 5.678)	0.039 (−5.504, 5.581)
≥6	−0.486 (−5.597, 4.626)	−0.856 (−0.605, 4.336)	−0.949 (−6.144, 4.247)
*P* for trend	0.845	0.948	0.713
Beneficial to gut microbiota	−0.172 (−3.880, 3.536)	−0.449 (−4.251, 3.353)	−0.396 (−4.203, 3.412)
Unfavorable to gut microbiota	−3.593 (−7.940, 0.754)	−3.542 (−7.906, 0.822)	−3.616 (−7.984, 0.751)
Tg (ng/mL)			
DI-GM	−1.180 (−3.495, 1.134)	−0.478 (−2.831, 1.876)	−0.395 (−2.752, 1.961)
DI-GM group			
0–3	Ref.	Ref.	Ref.
4	1.415 (−1.444, 4.275)	1.536 (−1.324, 4.397)	1.538 (−1.323, 4.0)
5	0.252 (−2.553, 3.056)	0.478 (−2.330, 3.286)	0.526 (−2.283, 3.335)
≥6	−1.337 (−3.932, 1.259)	−0.656 (−3.288, 1.976)	−0.595 (−3.229, 2.039)
*P* for trend	0.359	0.381	0.731
Beneficial to gut microbiota	−2.156 (−4.063, −0.248)	−1.578 (−3.530, 0.374)	−1.504 (−3.459, 0.451)
Unfavorable to gut microbiota	−0.250 (−2.438, 1.938)	−0.131 (−2.323, 2.061)	−0.180 (−2.374, 2.014)

DI-GM, dietary index for gut microbiota; TSH, thyroid-stimulating hormone; FT3, free triiodothyronine; FT4, free thyroxine; TT3, total triiodothyronine; TT4, total thyroxine; TgAb, thyroglobulin antibodies; Tg, thyroglobulin; TPOAb, thyroid peroxidase antibodies.

Model 1 was adjusted for age, sex, and ethnicity.

Model 2 was adjusted for age, sex, and ethnicity, education level, PIR, smoking status, alcohol consumption, and BMI.

Model 3 was adjusted for age, sex, and ethnicity, education level, PIR, smoking status, alcohol consumption, and BMI, urine iodine concentration, hypertension, and diabetes.

Subsequently, the DI-GM score was transformed into categorical variables, and in the fully adjusted model (Model 3), a negative association with FT3 (*β* = −0.043, 95% CI = −0.077, −0.010, *P* for trend = 0.010), FT4 (β = −0.011, 95% CI = −0.021, −0.002, *P* for trend = 0.027), and TT4 (β = −0.127, 95% CI = −0.237, −0.017, *P* for trend = 0.024) remained evident. In model 2, participants with a DI-GM score of 5 exhibited substantially higher TT3 levels (β = 1.699, 95% CI = 0.037 to 3.361). However, this relationship was no longer significant after further adjustment for additional covariates in model 3 (β = 1.582, 95% CI = −0.076, 3.239).

**Figure 2:**
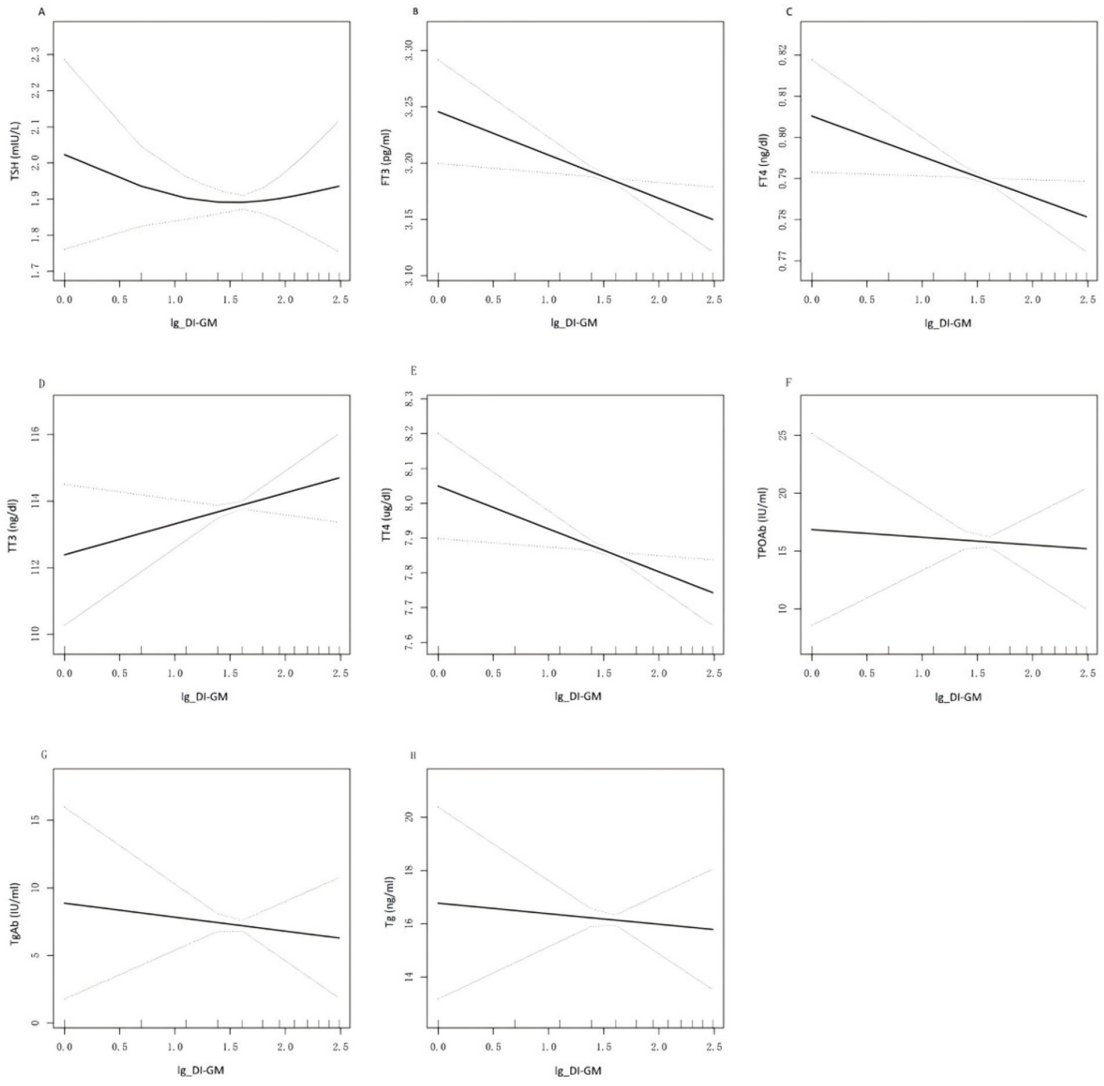
Relationship between DI-GM and thyroid function. **(A—H)** is a curve-fit plot of DI-GM versus thyroid function (TSH, FT3, FT4, TT3, TT4, TPOAb, TgAb, Tg). DI-GM dietary index for gut microbiota; TSH thyroid-stimulating hormone; FT3 free triiodothyronne; FT4 free thyroxine; TT3 total triiodothyronine; TT4 total thyroxine; TgAb thyroglobulin antibodies; Tg thyroglobulin; TPOAb thyroid peroxidase antibodies.

Smoothed curve fitting was used to visually assess the potential nonlinear correlation between DI-GM and thyroid function. As shown in Figure 2, after adjusting for all covariates, no nonlinear association was observed between DI-GM and FT3, FT4, TT3, TT4, TPOAb, TgAb, or Tg. However, a slight U-shaped pattern was observed in the relationship between TSH and DI-GM. The two-piecewise regression analysis revealed that DI-GM exceeded the threshold of 0.69, each unit increase in DI-GM was associated with 0.04 mIU/L increase in TSH levels. Conversely, below this threshold, each unit increase in DI-GM was associated with 0.55 mIU/L reduction in TSH levels. Threshold effect was close to the significance level, even if it did not reach it (likelihood ratio test *p* = 0.083) (Supplementary Table 2).

### 3.3 Subgroup analysis

**Figure 3:**
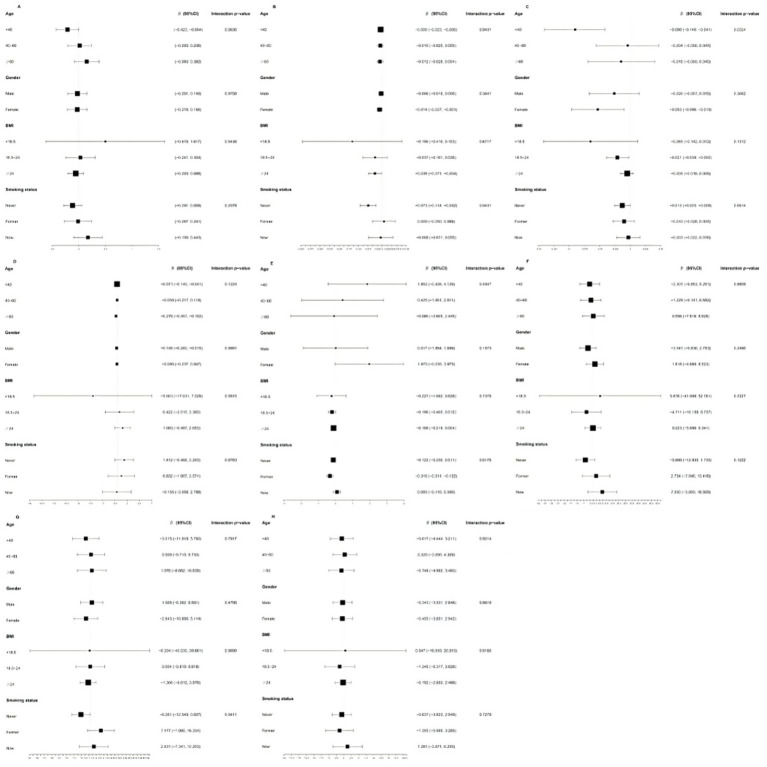
Subgroup analyses stratified by age, gender, BMI and smoking status. **(A)** thyroidstimulating hormone; **(B)** free triiodothyromne; **(C)** free thyroxine; **(D)** total triiodothyronine; **(E)** total thyroxine; **(F)** thyroid peroxidase antibodies; **(G)** thyroglobulin antibodies; **(H)** thyroglobulin.

Figure 3 shows the results of subgroup analyses stratified by age, sex, BMI, and smoking status. Age was identified as an interactive factor in the relationship between DI-GM and FT4 (p for interaction = 0.03). The negative association between DI-GM and FT4 was more than twice as strong in participants under 40 compared to older participants. Significant interactions were observed between DI-GM and FT3 (*p* for interaction = 0.04), TT4 (*p* for interaction = 0.02), and TgAb (*p* for interaction = 0.04) across different smoking statuses. Notably, a significant negative association with FT3 was observed only among participants who had never smoked (*β* = −0.073, 95% CI = −0.114, −0.032). A significant negative correlation with TT4 was observed only among former smokers (β = −0.316, 95% CI = −0.511, −0.122). For TgAb, although the differences were not statistically significant, a negative correlation was observed in participants who had never smoked (β = −6.261, 95% CI = −12.549, 0.027), whereas a positive relationship was found in former smokers (β = 7.177, 95% CI = −1.980, 16.334) and current smokers (β = 2.431, 95% CI = −7.341, 12.203). However, no significant interactions were identified regarding gender and BMI.

## 4 Discussion

This cross-sectional study examined the association between DI-GM and thyroid function using data from a large U.S. population from NHANES. The findings indicated a negative correlation between DI-GM and FT3, FT4, and TT4 levels. Even when DI-GM was analyzed as a categorical variable, participants with DI-GM ≥ 6 exhibited a significant negative association with FT3, FT4, and TT4 levels. No significant relationship was found between DI-GM and the other thyroid parameters. In addition, age appeared to influence the relationship between DI-GM and FT4 levels, whereas smoking status potentially moderated the correlations between DI-GM and FT3, TT4, and TgAb levels.

Previous studies have highlighted the importance of a healthy diet in supporting thyroid function. A cross-sectional study found that a higher Healthy Eating Index (HEI)-2010 score was associated with lower TT3 and FT3 levels in U.S. male adults (28). Another study reported that a 10-point increase in the HEI-2015 was associated with a 0.6% reduction in TT4 levels among U.S. adults (25). In addition, a 1-point increase in the relative Mediterranean diet score was associated with a 0.3% decrease in TT4 among women of reproductive age and a 0.5% reduction in TT3 levels among adult females (25). Zupo et al. (29) found that higher adherence to the Mediterranean diet, as assessed by the PREDIMED score, was negatively associated with FT3 and FT4 levels in a cohort of overweight and obese individuals from Apulia, Southern Italy. Liu et al. (30) reported that higher Composite Dietary Antioxidant Index scores, reflecting a diet rich in antioxidants, were associated with lower FT4 and TT4 levels. Ma and coworkers (31) identified a positive correlation between the dietary inflammatory index (DII) and TT4 levels in adult men based on NHANES data from 2007 to 2008. An analysis of NHANES 2007–2012 data revealed that individuals with higher DII scores experienced a significant increase in FT3 and TT4 levels in men and women (32). While all of these dietary indices have emphasized the impact of diet quality on thyroid functionality, their associations with gut microbiota diversity and richness indicators have been inconsistent. The novelty this study lies in introduction an new dietary indices, DI-GM, which not only measures the overall healthfulness of diet, but also focus on diet quality specifically related to gut microbiome health. Additionally, we focused on specific foods rather than broad food categories, which makes the research findings more easily translatable to clinical and public health decision-making. We found that DI-GM was negative correlation with FT3, FT4, and TT4 levels. This suggests that foods beneficial to gut microbiota diversity such as avocados, broccoli, chickpeas, coffee, cranberries, fermented dairy, fiber, green tea, soybeans, and whole grains, could likely be responsible for lowering thyroid hormone levels.

The gut microbiota reduces thyroid hormone levels through several pathways. First, it affects intestinal microelements uptake. Studies (15) suggest that a negative correlation between Lactobacillaceae and Bifidobacterium spp. with dietary iron. Iron deficiency can decrease the activity of thyroid iodine peroxidase, which plays a key role in thyroid hormone synthesis by catalyzing both the iodination of thyroglobulin and coupling of iodotyrosine molecules. A recent systematic review and meta-analysis (33) show that thyroid hormone levels are lower in patients with iron deficiency, especially in pregnant women. In addition, lactobacillus may increases the bioavailability of selenium. An animal study (34) has shown that all thyroids isolated from selenium-supplemented mice contained marginal vacuoles and a lower follicle area compared to the control group. The structural abnormality of the thyroid gland could decreased serum TT4 and FT4 levels. A cross-sectional study (35) revealed that the increased dietary selenium intake was negatively correlated with TT4 and TT4/TT3. Second, the microbiota can influence the conversion and storage of iodothyronines. Specifically, it can uncouple the sulfated glucuronide derivatives of iodothyronine through bacterial sulfate esterase or β-glucuronidase, thereby enhancing the reabsorption of thyroid hormones in enterohepatic circulation (36). Conversely, the inhibition of 5-deiodinase activity by the microbiota reduces the conversion of T4 to T3 and rT3 (12, 36). A study (37) utilizing a rat model found that deiodinase activity in the adult rat intestine was significantly lower than that observed in the rat fetus. This difference may be attributed to the inhibitory effects of resident intestinal microflora. Additionally, the microbiota can facilitate the binding of iodothyronines, acting as a reservoir (15) and consequently decreasing the circulating levels of thyroid hormones. Finally, microbiota can affect the thyroid hormone levels through their immunomodulatory effects. Studies (38) have demonstrated that specific strains of Bifidobacterium and Lactobacillus are pathogenic due to structural homology with the amino acid sequences of human TPO and Tg and thus can induce autoimmunity thyroid diseases through a cross-antigen-molecular mimicry mechanism. In addition, the DI-GM score reflects the production of SCFAs. By binding to G protein-coupled receptors, inhibiting the activity of histone deacetylase, maintaining intestinal mucosal barrier integrity, SCFAs can ameliorate both systemic inflammation (45), thereby reducing thyroid hormone levels. Studies have shown that the Systemic Inflammatory Response Index, as an indicator reflecting systemic inflammatory activity, was significant positive correlations with FT4 and TT4 levels (39).

Subgroup analyses and interaction tests were performed to explore potential differences between subgroups. The findings revealed substantial differences in the relationship between DI-GM and FT4 levels among different age groups. The negative association between DI-GM and FT4 was more than twice as strong in participants under 40 compared to older participants. As individuals age, they tend to become more health-conscious and make dietary improvements, which may contribute to higher DI-GM scores. This study suggests that participants with higher DI-GM scores exhibit a greater mean age. However, age-related microbiota plasticity plays a significant role, as younger individuals tend to have more adaptable microbiomes, thereby rendering dietary interventions more effective (22). In addition, the relationship between DI-GM and FT3, TT4, and TgAb levels varied among the groups with different smoking statuses. Among smokers, increasing the consumption of foods that support gut health did not appear to significantly lower FT3, TT4, and TgAb levels. A cross-sectional study previously reported that cigarette smoking was associated with slightly higher FT4 and FT3 levels, along with lower TSH levels (40). Smoking releases harmful substances, including nicotine, carbon monoxide, and thiocyanate. Notably, thiocyanate disrupts thyroid hormone synthesis by competitively inhibiting the uptake and organification of iodine in the gland (41). Smoking alters the composition of gut microbiota by promoting the growth of potentially pathogenic bacteria while decreasing the populations of beneficial bacteria (42, 43). Apart from smoking, smokers usually have other unhealthy lifestyle. This study suggests that smokers have lower DI-GM scores. In summary, smoking can diminish the positive impact of a gut-friendly diet on thyroid function.

This study’s strengths lie in its utilization of a large, nationally representative dataset, which enhances the reliability and generalizability of the results within the U.S. population. Additionally, this study is the first comprehensive exploration of the relationship between DI-GM and thyroid function, providing additional evidence supporting the efficacy of dietary interventions in improving thyroid function. However, several limitations must be acknowledged. First, this study employed a cross-sectional design, which limits the ability to infer causality. Longitudinal studies are needed to confirm the causal relationship between DI-GM and thyroid function. Second, reliance on 24-h dietary recall data for calculating DI-GM may introduce recall and self-reporting biases. Future studies should use more objective methods for dietary assessment. Mobile technology assisted dietary assessment may be a viable method that facilitates privacy, enables instant logging, and leverages standardized food databases (44). Third, the limitations of the DI-GM itself also deserve attention. The construction of the DI-GM was based on limited articles per food (19). Consequently, foods that have not been studied in relation to gut microbiota were not included in the index. Moreover, DI-GM provides an indirect estimation rather than a direct measurement of gut microbiota diversity (19). Future studies needed to be replicated in a dataset where direct gut microbiota diversity measures are available, and further explore the specific gut microbial profiles associated with DI-GM and their impact on thyroid hormone metabolism. At the same time, it will be necessary to supplement and update the DI-GM in the future as more studies emerge on the relationship between gut microbiota and dietary factors. This will enhance our understanding of the impact of diet on gut microbiota and its relationship with thyroid function. Fourth, the data in this study are derived from the U.S. population, which may limit applicability to populations in other countries, particularly in those with differing dietary culture and genetic backgrounds. Therefore, future studies should be conducted in populations from various races and regions to validate the generalizability and regional differences of the conclusions. In addition, participants with thyroid disease were excluded in this study. Future intervention trials are needed to assess whether dietary modifications to improve DI-GM scores can benefit individuals with thyroid disorders. Finally, despite accounting for numerous confounding variables, the potential influence of unknown or unmeasured factors, as well as residual confounding, cannot be entirely excluded.

In conclusion, DI-GM was negatively correlated with FT3, FT4, and TT4 levels. Participants with higher DI-GM scores, indicating a diet beneficial to gut microbiota health, tended to have lower FT3, FT4, and TT4 levels. In addition, our subgroup analyses revealed that age and smoking status influenced this association. However, larger prospective studies are required to validate our observational findings.

## Data Availability

The original contributions presented in the study are included in the article/supplementary material, further inquiries can be directed to the corresponding authors.
