# Supplementary material for: Association between the newly proposed dietary index for gut microbiota and thyroid function: NHANES 2007–2012
Source: Front Nutr. 2025 Jun 26;12:1602787. doi: 10.3389/fnut.2025.1602787 (PMC12240744; doi:10.3389/fnut.2025.1602787)
Supplement: Supplementary file 1 [file Table_1.docx]

| **STable1. Components of the DI-GM.** | | |
| --- | --- | --- |
| **Component** | **Included Foods within the Component** | **Scoring** |
| ****Beneficial to gut microbiota**** |  |  |
| Avocados | Avocados | For each component, a score of 1 if consumption at or above the sex-specific median, else 0 |
| Broccoli | Broccoli |  |
| Chickpea | Chickpeas |  |
| Coffee | Coffee |  |
| Cranberries | Cranberries |  |
| Fermented dairy | Yogurt, cheese, kefir, sour cream, buttermilk |  |
| Fiber | Not applicable |  |
| Soybean | Soy products—Soy milk, Tofu |  |
| Whole grains | Grains defined as whole grains, containing the entire grain kernel―the bran, germ, and endosperm |  |
| ****Unfavorable to gut microbiota**** |  |  |
| High-fat diet (% energy) | Not applicable | 0 if consumption at or above 40% energy from fat, else 1 For each remaining component, a score of 0 if consumption at or above the sex-specific median, else 1 |
| Processed meat | Frankfurters, sausages, corned beef, and luncheon meat that are made from beef, pork, or poultry |  |
| Red meat | Beef, veal, pork, lamb, and game meat; excludes organ meat and cured meat |  |
| Refined grains | Refined grains that do not contain all of the components of the entire grain kernel |  |
