# Supplementary material for: Association between the newly proposed dietary index for gut microbiota and thyroid function: NHANES 2007–2012
Source: Front Nutr. 2025 Jun 26;12:1602787. doi: 10.3389/fnut.2025.1602787 (PMC12240744; doi:10.3389/fnut.2025.1602787)
Supplement: Supplementary file 2 [file Table_2.docx]

| STable 2. Threshold effect analysis | | | | | |
| --- | --- | --- | --- | --- | --- |
|  | Continuous | Inflection point（K） | <K | >K | Log likelihood ratio |
| TSH | -0.03 (-0.16, 0.10) 0.6726 | 0.69 | -0.55 (-1.15, 0.05) 0.0749 | 0.04 (-0.11, 0.19) 0.6267 | 0.083 |
| FT3 | -0.04 (-0.07, -0.01) 0.0121 | 0.69 | 0.08 (-0.06, 0.22) 0.2628 | -0.05 (-0.09, -0.02) 0.0025 | 0.089 |
| FT4 | -0.01 (-0.02, -0.00) 0.0309 | 0.69 | -0.01 (-0.05, 0.03) 0.6327 | -0.01 (-0.02, 0.00) 0.0614 | 0.988 |
| TT3 | 0.93 (-0.46, 2.32) 0.1899 | 1.39 | 1.14 (-1.32, 3.60) 0.3626 | 0.71 (-1.82, 3.24) 0.5813 | 0.838 |
| TT4 | -0.12 (-0.22, -0.02) 0.0144 | 1.61 | -0.15 (-0.29, -0.02) 0.0270 | -0.04 (-0.31, 0.22) 0.7434 | 0.520 |
| TPOAb | -0.67 (-6.09, 4.76) 0.8097 | 1.61 | 2.20 (-5.31, 9.72) 0.5651 | -8.04 (-22.43, 6.36) 0.2738 | 0.278 |
| TgAb | -1.04 (-5.69, 3.62) 0.6625 | 1.61 | 0.52 (-5.92, 6.96) 0.8741 | -5.03 (-17.37, 7.31) 0.4243 | 0.492 |
| Tg | -0.40 (-2.75, 1.96) 0.7424 | 1.1 | 1.93 (-4.08, 7.94) 0.5292 | -1.32 (-4.55, 1.90) 0.4221 | 0.409 |

TSH: thyroid-stimulating hormone; FT3: free triiodothyronine; FT4: free thyroxine; TT3: total triiodothyronine; TT4: total thyroxine; TgAb: thyroglobulin antibodies; Tg: thyroglobulin; TPOAb: thyroid peroxidase antibodies.
